# The Potential Therapeutic Use of Agarwood for Diabetes: A Scoping Review

**DOI:** 10.3390/ph17111548

**Published:** 2024-11-18

**Authors:** Mohammad Adi Mohammad Fadzil, Norhashimah Abu Seman, Aswir Abd Rashed

**Affiliations:** Nutrition, Metabolism and Cardiovascular Research Centre (NMCRC), Institute for Medical Research (IMR), National Institutes of Health (NIH), Ministry of Health Malaysia (MOH), No. 1, Jalan Setia Murni U13/52, Seksyen U13 Setia Alam, Shah Alam 40170, Malaysia; adifadzil@moh.gov.my (M.A.M.F.); nor.hashimah@moh.gov.my (N.A.S.)

**Keywords:** agarwood, diabetic properties, phytochemicals, therapeutic potential

## Abstract

Introduction: In 2019, 9.3% (463 million) of adults worldwide had diabetes, according to the International Diabetes Federation (IDF). By 2030, the number will rise to 10.2% (578 million) and 10.9% (700 million) by 2045 if effective prevention methods are not implemented. Agarwood is a pathological product and valuable plant due to its numerous medicinal properties, and it is used as an essential ingredient in medicine. Therefore, we conducted this review to determine agarwood’s potential health benefit effect on type 2 diabetes. Results and Discussion: Although no clinical trials were found, the evidence from in vitro and in vivo studies is promising. Agarwood has shown the ability to reduce the activity of α-glucosidase, α-amylase, and lipase, promote adiponectin secretion during adipogenesis, and reduce oxidative stress. Animal studies elucidated hypoglycaemic, antidyslipidemia, anti-obesity, and organ protective effects from agarwood. Materials and Methods: Original articles were searched in three databases (PubMed, Scopus, and Cochrane Library) using the medical subject heading (MeSH) term “diabetes” crossed with the term “agarwood” from 2008 to 2024. Synonyms and relevant search terms were also searched. Conclusions: This effect underscores the need for further research and the potential for groundbreaking discoveries in the field of diabetes treatment.

## 1. Introduction

Agarwood, also known as eaglewood, *oud*, *oudh*, aloeswood, *karas*, and *gaharu*, is a type of resinous, fragrant heartwood produced by the tree species of the family Thymelaeaceae. Typically, agarwood is obtained or harvested from the genera *Aquilaria* and *Gyrinops* [1]. Agarwood is a secondary metabolite produced as a defence mechanism by agarwood-producing species upon encountering injuries or infections. The dark and aromatic resin accumulates in the heartwood of the trees [1]. However, not all genus *Aquilaria* or *Gyrinops* trees can produce agarwood. There are only 17 recognised species around the world that have been recorded as being able to produce agarwood [2]. These species are commonly found in Southeast Asia, China, and India. *A. malaccensis*, *A. sinensis*, *A. rugosa*, *A. filaria*, *A. subintegra*, *A. crassna, A. agallocha*, and *A. beccariana* are the common species used for the harvesting of agarwood [3].

Due to its significant economic nontimber potential and limited availability, agarwood is highly sought after, especially in the Asia and Middle East markets. A recent review by the Convention of International Trade in Endangered Species of Wild Fauna and Flora (CITES) found that overharvesting has destroyed the agarwood-producing species’ natural habitat. Thus, technological advancement has been made in producing artificial agarwood with physical and medicinal properties on par with natural agarwood [4]. The use of agarwood is deeply rooted in many traditions for various purposes, as its application is recorded in historical and religious scriptures such as those of the Sahih Muslims and the Ayurveda [1]. Agarwood has been recorded in the literature as having various pharmacological benefits such as antibacterial, anticancer, anti-inflammation, antioxidant, analgesic, cough depressant, asthma reliever, anti-ageing, cardioprotective, and anti-depression effects [5,6,7,8,9,10,11,12]. The beneficial health properties are due to the various active compounds that have been identified through phytochemical studies, such as chromone derivatives, terpenoids, flavonoids, benzophenones, lignans, benzenoid derivatives, phenolic compounds, triterpenes, steroids, and other chemical compounds [1].

Diabetes mellitus is a complex metabolic disorder characterised by a chronic elevated sugar level in the bloodstream, a phenomenon commonly termed hyperglycaemia, which is the hallmark of the disease. Although there are more types of diabetes occurring, type 1 diabetes and type 2 diabetes are the two primary variants, making up 5% and 90% of diabetes cases worldwide [13].

The epidemiologic trend of diabetes is worrying, as the International Diabetes Federation reported that the global number of diabetes incidence is expected to increase from 537 million in the year 2021 to 783 million in the year 2045 [14]. In the year 2021 alone, 6.7 million mortalities of young adults and the elderly were caused by diabetes and its related diseases. The economic burden of diabetes is also worrisome, as the global cost of treating the disease is expected to reach 1.05 trillion USD by 2045 [14].

Diabetes is also known to be a polygenic disease caused by a multifactorial interaction of environmental factors and genetic variations. However, the common themes are that nutrition transition, rapid urbanisation, rising cases of obesity, and modern sedentary lifestyles lead to an increased risk of diabetes, especially type 2 diabetes [15]. Ultimately, the most preferred and effective therapeutic and management approaches to diabetes are lifestyle modifications involving diet changes, active lifestyles, and pharmacological interventions [16]. Interventions involving individualised precision and personalised strategy are becoming more popular nowadays, aiming to meet glycaemic goals.

Up to date, many oral conventional drugs are available to reduce glucose levels, such as sulfonylureas, meglitinides, biguanides, thiazolidinediones, α-glucosidase inhibitors, α-amylase inhibitors, dipeptidyl peptidase-4 (DPP-4) inhibitors, sodium-glucose cotransporter-2 (SGLT2) inhibitors, glucagon-like peptide-1 (GLP-1) agonist, and cycloset [16,17]. Among these conventional drugs, α-glucosidase and α-amylase inhibitors are designed to delay or prevent blood glucose spiking by inhibiting the hydrolysation of carbohydrates into glucose [18,19,20]. Examples include acarbose, miglitol, and voglibose, commonly prescribed for diabetes management. Other than that, it is well established that targeting and preventing obesity will result in a better prognosis and prevention of type 2 diabetes. Thus, lipase inhibitors are also commonly prescribed drugs aimed at disrupting fat absorption from food, resulting in reduced weight gain [21]. An example of a conventional drug is orlistat. However, these conventional drugs often come with side effects such as flatulence, oily stools, and gastrointestinal complications, leading to poor compliance and efficacy [18,19,20,22].

Due to their possible health advantages and minimal side effects, functional foods are becoming more popular for managing diabetes [23]. Compared to traditional diabetic treatments, functional foods are frequently more affordable and readily available, making them a viable choice for many people [24]. Agarwood can be potentially utilised as a functional food due to its high content of bioactive chemicals, providing an alternative to conventional diabetic treatments. Many studies have reported the anti-diabetic properties of agarwood. Therefore, this review will evaluate agarwood’s potential as a supplementary or alternative agent for managing diabetes disease.

## 2. Materials and Methods

The study question was constructed based on the PICO framework [25]. The PICO framework stands for Population or subjects, Intervention or exposure, Comparison or control, and Outcome or benefits. The population/subjects investigated are individuals or animals with diabetes. The intervention is the application of agarwood. The comparison or control is the conventional diabetes treatment. The study aims to elucidate agarwood plants’ potential use and mechanism in managing diabetes.

The inclusion criteria were as follows: (1) Primary studies that evaluated the efficacy of agarwood against diabetes. (2) Peer-reviewed studies with an available full text. (3) Studies published in the English language. (4) Included studies of any geographical origin from 2008–2024 (15 years). The exclusion criteria were as follows: (1) Duplicate publications. (2) Studies published in a non-English language. (3) Review articles, protocol papers, letters to editors, and short communications.

**Figure 1:**
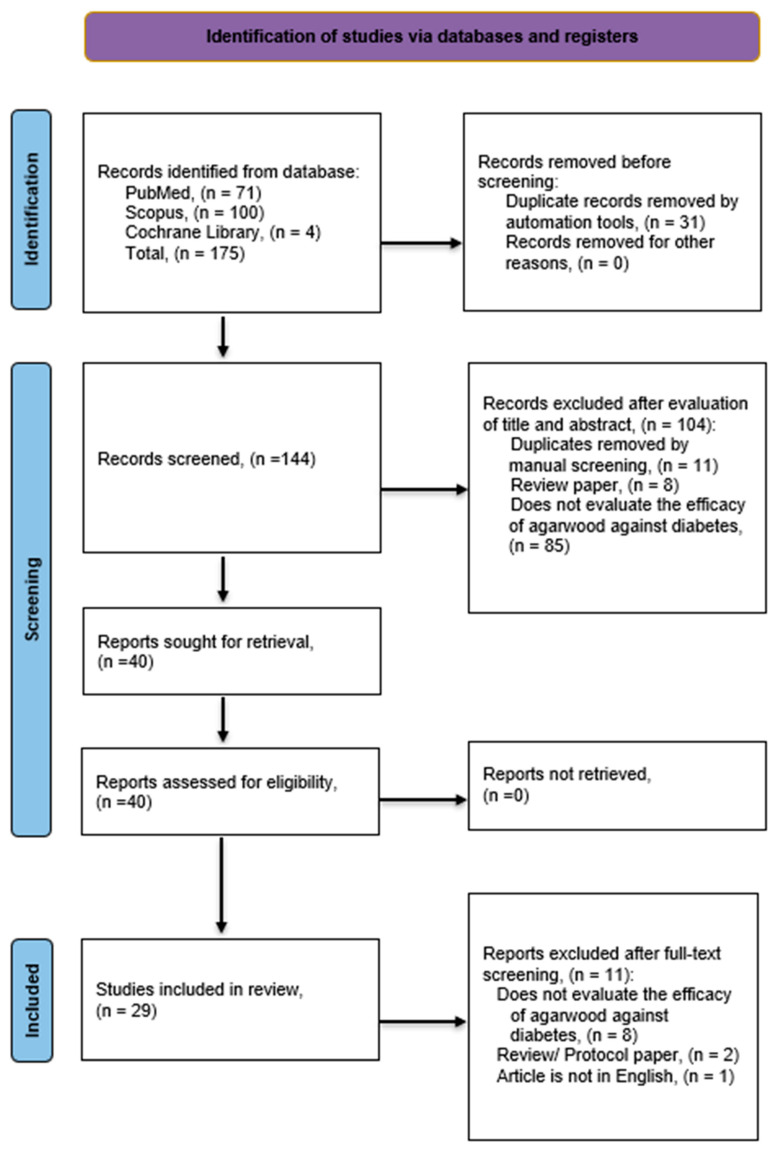
Flowchart of the Search Strategy.

Original articles were comprehensively searched in three databases (PubMed, Scopus, and Cochrane Library) using the medical subject heading (MeSH) term “diabetes” crossed with the term “agarwood.” Search terms also included synonyms and relevant keywords for diabetes and agarwood. The search terms were then connected using Boolean operators “OR” and “AND” before being subjected to database browsing. The search terms for each database are shown in the Supplementary Materials. The last search was carried out in February 2024. The decision to focus on studies published from 2008 onwards was based on the aim of capturing the most recent and relevant research developments in the field over the past 15 years. All database hits were subjected to automated duplicate screening using the citation manager EndNote X9 (Clarivate Analytics, Philadelphia, PA, USA). After that, the title and abstract of the records were screened manually for duplicates or illegibility. Finally, the remaining articles’ full texts were assessed per the stated criteria. Mohammad Adi Mohammad Fadzil (MAMF) and Aswir Abd Rashed (AAR) were involved in the selection process following the eligibility criteria. Contradicting assessments were resolved through discussions. Flowcharts were constructed using the “Preferred Reporting Items for Systematic Reviews and Meta-Analyses” (PRISMA) [26]. The detailed process of the search strategy is elucidated using a flowchart in Figure 1.

## 3. Results

The search resulted in 175 articles produced with a refined search based on the availability of the full text, peer-reviewed articles, and library collection access. Upon further assessment, only 29 full-text articles were relevant and included for the final review (Table 1 and Table 2). All the related articles were printed out for further evidence-based assessment to explore agarwood’s effectiveness as a potential anti-diabetic agent. Overall, 22 studies were conducted in vitro [27,28,29,30,31,32,33,34,35,36,37,38,39,40,41,42,43,44,45,46,47] and 7 were in vivo studies [48,49,50,51,52,53,54]. Studies were conducted using agarwood plants obtained from various countries. Specimens from Thailand, Indonesia, Malaysia, Korea, Laos, Vietnam, India, Algeria, and China featured in most studies. Various agarwood-producing plant species were studied for their anti-diabetic potential. A total of 11 studies were conducted on *A. sinensis* [29,37,39,40,41,46,47,48,49,53,55], 7 studies on *A. malaccensis* [27,28,33,36,45,47,54], and 5 studies on *A. crassna* [32,38,39,44,50]. Other agarwood-producing plants studied are *A. filaria*, *A. subintegra*, *A. agallocha*, and *G. versteegii* [27,30,31,33,42,43,47,51,52], including two unspecified *Aquilaria* species [34,35]. Most studies were conducted on either the agarwood itself or the leaves; 14 studies were conducted using agarwood [28,30,31,34,35,37,39,40,41,42,43,44,45,51,52] whereas 13 studies were conducted using the leaves [27,29,31,33,36,38,46,47,48,49,50,53,55]. Other studies used different parts of the agarwood-producing plants, such as the stem bark, fruit bark, and branches. Three studies were conducted to investigate the potential synergistic effects of agarwood and other plants [36,52,54]. Ethanol was the most used extraction solvent, where it was used in 12 studies [36,37,38,39,40,41,42,43,44,46,47,53]. The second most used solvent was methanol extraction, used in nine studies [30,31,32,33,45,48,49,54,55] and the third was water extraction, used in seven studies [27,28,47,48,50,51,52]. Other extraction solvents used were hexane, ethyl ether, dichloromethane, and acetone [29,30,33,34,35,47].

## 4. Discussion

### 4.1. In Vitro Anti-Diabetic Findings of Agarwood

**Figure 2:**
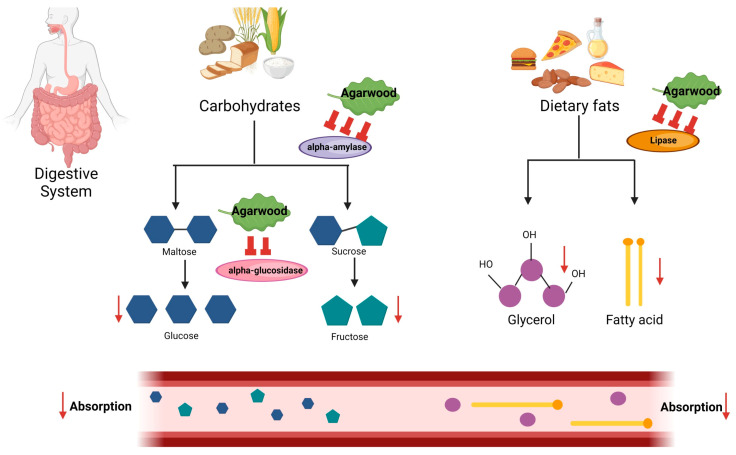
Overview of in vitro studies on the anti-diabetic mechanisms of agarwood.

In vitro studies on the anti-diabetic mechanisms of agarwood are mainly characterised by the plant’s potent efficacy in inhibiting enzymes associated with carbohydrate metabolism, exhibiting lipase inhibitory activity, and increasing the production of adiponectin as well as oxidative stress. Figure 2 shows the overview of the in vitro anti-diabetic mechanism of agarwood on carbohydrates and dietary fats in the intestine.

#### 4.1.1. α-Glucosidase Inhibition

α-glucosidase is an enzyme involved in the breaking down of oligosaccharides and disaccharides into glucose [56]. Excess glucose rapidly absorbed and retained in the bloodstream, especially after meals, is commonly described as postprandial hyperglycaemia [57]. The conventional drugs prescribed are α-glucosidase inhibitors, such as acarbose and miglitol; they are associated with side effects, such as gastrointestinal discomfort and long-term tolerance [58,59]. Therefore, alternative and complementary natural products with minimal side effects are needed to inhibit α-glucosidase to manage type 2 diabetes.

Several studies have reported promising α-glucosidase inhibitors in various compounds extracted from agarwood, particularly polyphenols, sesquiterpenes, chromones, and benzophenone O-glycosides. One of the most studied compounds is the 2-(2-phenylmethyl) chromone due to its strong α-glucosidase inhibition. In a study using *A. filaria* agarwood from Indonesia, 6,7-dihydroxy-2-(2-phenyl ethyl) chromone was identified as the most potent chromone derivative to inhibit the α-glucosidase activity with an IC_50_ value of 2.256 ± 0.085 μg/mL, surpassing the potency of the conventional drug, acarbose, and comparable to genistein with IC_50_ values of 479.939 ± 2.130 μg/mL and 2.243 ± 0.027 μg/mL, respectively [42]. Another study conducted using *A. sinensis* agarwood from China reported 6,7-dihydroxy-2-[2-(4-methoxyphenyl) ethyl] chromone as the most potent derivative from that agarwood species with an IC_50_ value of 15.655 μg/mL [40]. Besides chromones, sesquiterpenoid derivatives are among the primary compounds isolated from agarwood for their bioactive potential. In a study of sesquiterpenoid derivatives isolated from agarwood obtained from Thailand, the most potent inhibitor of α-glucosidase was identified as Aquilarene D with an IC_50_ value of 57.460 μg/mL, also surpassing the potency of the conventional drug acarbose with an IC_50_ value of 781.176 μg/mL [35]. For *A. filaria* agarwood, the most potent sesquiterpenes against α-glucosidase activity were determined as guaianolide, with an IC50 value of 53.332 ± 2.273 μg/mL [43]. Also in 2019, screening for new sesquiterpenoids from agarwood found a newly discovered zizaane-type sesquiterpenoid named agarozizanol E with an IC_50_ value of 27.308 μg/mL, and it was found to act as an uncompetitive inhibitor due to the compound’s ability to modify the active site of the α-glucosidase enzyme. The authors emphasised that uncompetitive inhibitors are preferred in drug discovery due to their high efficacy in living models [34].

Other than these types of compounds, benzophenone O-glycosides are also commonly studied in agarwood for their α-glucosidase inhibition potential. 4,4′,6-O-trihydroxylbenzophenone 2-O-α-D-mannopyranosyl-(1-4)-α-L-rhamnoside, isolated from the leaves of *A. sinensis*, showed notable inhibitory activity with an IC_50_ value of 93.713 ± 7.721 μg/mL, adding to the diverse array of bioactive compounds found in agarwood [29]. Mangiferin is a significant polyphenolic commonly identified as the active compound responsible for the anti-α-glucosidase properties of agarwood. Mangiferin content was determined to make up 2.1130% of the dried leaf powder [38]. Mangiferin isolated from the leaves of *A. sinensis* and *A. crassna* showed IC_50_ inhibition of α-glucosidase at 126.5 ± 17.8 ug/mL and 571.4 ± 4.4 ug/mL, respectively [38,46]. However, a study conducted using *A. crassna* leaves from Vietnam revealed genkwanin, another polyphenol, to be the most potent α-glucosidase inhibitor with an IC_50_ value of 6.822 ug/mL [32].

Studies have also focused on comparing the α-glucosidase inhibition between wild and artificially induced agarwood. Artificial technology aims at a faster and more sustainable production of agarwood. For instance, wild agarwood demonstrated an IC_50_ value of 156.4 μg/mL, compared to 292.0 μg/mL for artificially produced agarwood [39]. A more recent study in 2023 reported similar trends, with wild agarwood showing an IC_50_ value of 147.2 μg/mL, compared to 211.9 μg/mL for the artificially induced variant [37]. These studies elucidated that although wild agarwood is still superior in terms of α-glucosidase inhibition, this signals that artificially produced agarwood technology is improving and approaching the efficacy of natural agarwood.

Much research has been carried out involving the optimisation of using different solvents, drying methods, and parts of the plant to achieve the highest α-glucosidase inhibition. For instance, acetone is the preferred extraction solvent for *Gyrinops versteegii* from Indonesia, showing the highest potency against α-glucosidase compared to other solvents [30]. Also, the leaf extracts of *Gyrinops* versteegii performed better than the fruits and barks of the plant [31]. On the other hand, an ethanolic extract of *A. crassna* leaves from Thailand reported an IC_50_ value of 184.0 ± 3.2 ug/mL against α-glucosidase [38]. Interestingly, another study reported a much higher potency from *A. crassna* leaves extracted using boiling water with an IC_50_ value of 36.3 ± 2.3 μg/mL [50]. In a Malaysian study, leaves from *A. malaccensis*, *A. subintegra*, and *A. sinensis* were compared for their α-glucosidase inhibition using different drying methods and solvents. The study found that 70% ethanol extraction from oven-dried *A. malaccensis* leaves resulted in the highest potency, with an IC_50_ value of 0.13 μg/mL, almost five times more potent than the acarbose control [47]. These various studies prove that extraction parameters significantly influence the potency of the bioactive compounds from agarwood against α-glucosidase.

Interestingly, a synergistic effect between C. *burmannii* bark and *A. malaccensis* leaves on α-glucosidase inhibition was reported [36]. A 750:250 μg/mL *C. burmannii* and *A. malaccensis* extract combination showed higher inhibition at 96.368 ± 0.00870% compared to a pure 1000 μg/mL *A. malaccensis* extract at 83.133 ± 0.00870% inhibition. In the same study, in silico molecular docking revealed that palustrol, a secondary metabolite from A. malaccensis, was able to bind strongly to the receptor-ligand of the human maltase-glucoamylase receptor. The binding affinity of palustrol was −10.2 kcal/mol, whereas the acarbose control was −8.1 kcal/mol [36]. These findings highlight the potential of agarwood and its active compounds in the discovery and development of effective α-glucosidase inhibitors for diabetes management.

#### 4.1.2. Lipase Inhibition

Lipase is one of the enzymes involved in the function of the gastrointestinal tract. Lipase breaks down into triacylglycerides, monoacylglycerols, and free fatty acids [60]. Inhibiting lipase is a strategy to manage obesity and type 2 diabetes, as it reduces lipid levels and relieves stress on pancreatic β-cells, improving insulin regulation. However, synthetic inhibitors like orlistat can cause side effects, making the search for natural alternatives important in managing these conditions [61].

In a study conducted in Malaysia, two *Aquilaria* spp. were collected to investigate their potential to inhibit lipase activity [33]. The leaves and bark of *A. subintegra* and *A. malaccensis* were collected, dried, and grided for homogenisation. Then, the leaves and bark of *A. subintegra* and *A. malaccensis* were individually extracted using macerations for 72 h using three different solvents—hexane, dichloromethane, and methanol. In total, there were 12 crude extracts of the *Aquilaria* spp. The anti-lipase activity was measured by employing a colorimetric assay using porcine pancreatic lipase and olive oil as the natural substrate. The positive control used was lipase enzyme and substrate without the crude extracts. Lipase activity in the positive control was measured at 0.753 U/mL ± 0.037. Then, 100 μg/mL of the crude extracts were screened for inhibitory potential. Among the 12 crude extracts, *A. malaccensis* bark extracted using dichloromethane showed the highest inhibitory activity at 91% inhibition, *p* < 0.01 (0.065 U/mL ± 0.147). As for *A. subintegra*, the highest inhibition was observed from its leaves extracted using hexane at 54% inhibition, *p* < 0.01 (0.348 U/mL ± 0.077). Phytochemical screening was conducted, and all extracts were found to have flavonoids, steroids, and terpenoids. The authors concluded that anti-lipase activity may be attributed to the polyphenol activity [33]. This study proves that different parts and extraction solvents play an important role in determining the inhibition of lipase activity using agarwood.

In another study from Malaysia, the roles of particle size and the kinetic behaviour of lipase inhibition using *A. subintegra* and *A. malaccensis* were studied [27]. *A. subintegra* and *A. malaccensis* leaves were collected, dried, milled, and sieved into five separate particle sizes ranging from 250 μm to 1000 μm. The powdered leaves were macerated using distilled water for 24 h before being sonicated for 30 min at 60 °C. The crude extracts were obtained using hydrodistillation. After that, the total polyphenols and total flavonoids were determined, and a kinetic study of lipase inhibition was carried out. The study found that crude extracts extracted using the smallest particle size (250 μm) contained the highest concentration of the gallic acid equivalent of the total polyphenols and quercetin equivalent of the total flavonoids. The gallic acid equivalent concentrations for A. *subintegra* and *A. malaccensis* using a 250 μm particle size are 101,273.0 μg/mL and 89,991.0 μg/mL, respectively. The quercetin equivalent concentrations for *A. subintegra* and *A. malaccensis* using a 250 μm particle size are 37.0 μg/mL and 29.0 μg/mL, respectively. Also worth noting is that the gallic acid and quercetin equivalents were 10% and 20% higher in *A. subintegra* compared to *A. malaccensis*. Similarly, the highest percentage inhibition of lipase activity was recorded at 82% using 1 mL of *A. subintegra* crude extracts from a 250 μm particle size powder. These findings support that a higher surface area of powder allows for the better extraction of polyphenols and flavonoids, leading to a higher lipase inhibition potential. Using Lineweaver–Burk plots, the mode of inhibition exhibited by *A. malaccensis* and *A. subintegra* leaf crude extract was described as a mixed inhibition that closely mimics noncompetitive binding. The authors described the possibility of an allosteric binding site on the lipase enzyme. This reaction allows the *Aquilaria* spp. extracts to bind to free lipase enzymes and to the lipase–fat complex. The study concluded that both the lipase–*Aquilaria* spp. complex and the lipase–*Aquilaria* spp.–fat complex prevent the formation of fatty acid products [27].

#### 4.1.3. α-Amylase Inhibition

α-amylase breaks down digestive starches into simpler sugars to be absorbed into the bloodstream, causing blood glucose levels to increase [62]. Thus, it is a therapeutic strategy to lower complex sugar breakdown by inhibiting α-amylase activity to aid in the gradual release of glucose into the blood [63]. Therefore, screening for anti-α-amylase properties is common in testing natural products’ anti-diabetic potential.

In India, *A. malaccensis* resinous agarwood was obtained by naturally infesting the tree with insects. The produced resinous agarwood was tested for α-amylase inhibitory activity. Tiny chips of the resinous agarwood were subjected to extraction using the Clevenger apparatus to obtain agarwood essential oils. At 60 μL/mL, the essential oil showed 85.90 ± 0.0018% inhibition, better than the conventional drug control, acarbose, at 68.42 ± 0.0080%. Following this, the IC_50_ was determined for the agarwood essential oil at 30.78 ± 0.0018 μL/mL, whereas the IC_50_ for acarbose was determined at 44.77 ± 0.0006 μL/mL [28]. These promising results unequivocally demonstrate the potential use of agarwood essential oil as an alternative strategy to inhibit α-amylase activity, marking a significant advancement in the field of pharmacology and herbal medicine.

Next, the α-amylase inhibition potential of *A. malaccensis* leaves and *Cinnamomum burmannii* barks was investigated [36]. The plant’s materials were obtained from Indonesia. Ethanolic extraction was employed using the maceration technique. A sample of 0.25 mL of 1000 μg/mL of the *A. malaccensis* leaf extract showed 77.098 ± 0.521% inhibition towards α-amylase, whereas 1000 μg/mL *Cinnamomum burmannii* barks showed 79.397 ± 0.099% inhibition. The results are comparable to the acarbose control at 78.143 ± 0.099% inhibition. It is worth noting that both plants showed synergistic effects in inhibiting α-amylase. A combination of 75% *Cinnamomum burmannii* and 25% *A. malaccensis* showed the highest inhibition at 86.366 ± 0.451% [36]. These results support the polyherbal use of agarwood and other herbal products for synergistic effects.

#### 4.1.4. Adiponectin Production

Adiponectin is a hormone produced by white adipose that has been linked to anti-obesity, anti-diabetes, and anti-cardiovascular diseases [45]. Studies have shown that adiponectin activates the Peroxisome Proliferator-Activated Receptor Alpha (PPAR-α) and AMPK pathways, which enhance insulin sensitivity and fat metabolism and reduce serum glucose and triglyceride levels. However, long-term use of synthetic PPARγ agonists, like thiazolidinediones, can cause side effects, emphasising the need for safer alternatives [64,65,66,67,68,69]. This underscores the urgent need for research to discover alternatives that can minimise the risk of the side effects of synthetic PPARγ modulators.

In a study by Ahn et al. (2019), the bioactive compounds of phenyethylchromone were isolated from agarwood chips of *A. malaccensis* [45]. The extracts, obtained by reflux in 70% methanol, were further fractionated using multiple solvents—water, diethyl ether, ethyl acetate, and n-butanol. Later, the respective chromone compounds were isolated using a chromatographic technique. Using a human bone marrow mesenchymal stem cell model, each of the obtained chromone compounds was tested for their effects on adiponectin production during adipogenesis. The compounds 6-methoxy-2-(2-phenylethyl) chromone and 7-methoxy-2-(2-phenylethyl) chromone were identified as the most potent promoters of adiponectin production, with EC_50_ values at 4.541 μg/mL, and 5.690 μg/mL, respectively, ensuring the reliability of the results. Further investigation found that the increased adiponectin was due to the activation of the PPARγ. Thus, Ahn et al. (2019) reported that *A. malaccensis* contains bioactive phenyethylchromone that exhibited PPARγ partial agonism, causing increased adiponectin production [45]. This discovery holds significant promise for diabetic patients, as increased adiponectin levels promote insulin sensitivity and decreased inflammation in diabetic patients.

#### 4.1.5. Antioxidant Potential

Various studies have investigated the antioxidant capacity of agarwood extracts and essential oils using different assays such as DPPH, ABTS, and FRAP radical scavenging activities. First, methanol, methanol-water, and acetone of inoculated *G. versteegii* wood were tested using a DPPH radical scavenging assay. Results showed that all the extracts contained a promising antioxidant value, with the best antioxidant value being acetone extract with an IC_50_ value of 65.62 μg/mL [30].

Ma et al. (2021) conducted a comparative study on the antioxidant potential of various agarwoods [39]. The study found that the most potent IC_50_ values of DPPH and ABTS free radicals were reported in wild *A. sinensis* at 307.0 μg/mL and 236.8 μg/mL, respectively. An 18-month inoculated *A. sinensis* was reported as the best antioxidant artificial agarwood with the IC_50_ values of the DPPH and ABTS free radicals at 390.2 μg/mL and 447.2 μg/mL, respectively. Similar studies were conducted in 2023 by Ma et al. to compare the antioxidant potential of artificial and wild agarwood [37]. As a result, the IC_50_ values for DPPH free radical scavenging by artificial and wild agarwood were 187.3 μg/mL and 151.5 μg/mL, respectively. Furthermore, the IC_50_ values for the ABTS free radical scavenging assays for artificial agarwood and wild agarwood were 60.2 μg/mL and 56.3 μg/mL, respectively. Statistical analysis showed that the difference between artificial and wild agarwood is not significant (*p* > 0.05) [37]. Thus, regarding antioxidant potential, artificial technology in producing agarwood is improving and approaching naturally produced agarwood.

A study in Malaysia investigated the DPPH activity of *A. malaccensis*, *A. subintegra*, and *A. sinensis* leaf extracts using various extraction solvents and drying techniques [47]. The DPPH activity assay revealed that oven-dried *A. malaccensis* leaves extracted using 70% ethanol have the most potent antioxidant capacities with an IC_50_ value of 33.60 ug/mL. Correlation studies showed a positive correlation between the total polyphenols content and DPPH activity (r = 0.796, *p* < 0.05) [47]. This correlation might signal that the antioxidant capacity of agarwood leaf extracts is due to the rich polyphenol content.

*A. crassna* leaf crude extracts and mangiferin isolated from the extracts were tested for antioxidant activity [38]. A DPPH scavenging assay using *A. crassna* leaf extracts and mangiferin reported IC_50_ values of 21.54 ± 0.17 ug/mL and 0.64 ± 0.005 ug/mL, respectively. The DPPH activity of mangiferin isolated from *A. crassna* proved to be more potent than quercetin with an IC_50_ value of 3.46 ± 0.09 ug/mL. The IC_50_ values for a nitric oxide radical scavenging assay using *A. crassna* leaf extracts and mangiferin were recorded at 79.13 ± 0.74 ug/mL and 40.55 ± 0.17 ug/mL, respectively. Next, the IC_50_ values for a superoxide radical scavenging assay using *A. crassna* leaf extracts and mangiferin were recorded at 278.12 ± 4.29 ug/mL and 105.49 ± 1.28 ug/mL, respectively. Thus, this study demonstrates that *A. crassna* leaf extracts exhibit potent antioxidant activity. Mangiferin is suggested as the active compound contributing to the antioxidant potential, outperforming the crude leaf extracts in various radical scavenging assays and quercetin in the DPPH scavenging assays [38].

A study compared the antioxidant DPPH radical scavenging potential of fruit bark, stem bark, and the leaves of an agarwood-producing plant, *G. versteegii* [31]. The study also compared the antioxidant potential between healthy and inoculated *G. versteegii*. As a result, the inoculated specimens reported higher DPPH radical scavenging potential, with the most potent being the inoculated leaves of *G. versteegii* extracts with an IC_50_ value of 32.89 ± 2.7 ug/mL. Thus, this study showed that *G. versteegii* leaves possess the most potent antioxidant potential compared to other parts of the plant, with enhanced bioactivity due to inoculation [31]. Gogoi et al. (2023) determined the antioxidant potential of *A. malaccensis* using its essential oil [28]. The *A. malaccensis* essential oil reported an IC_50_ value of 40.14 ± 0.0192 μL/mL using the DPPH free radical scavenging assay. An ABTS scavenging assay of the agarwood essential oil reported an IC_50_ value of 76.95 ± 0.0090 μL/mL, whereas the IC_50_ value for a metal chelating assay was reported at 26.96 ± 0.0244 μL/mL [28]. These findings further emphasised the antioxidant potential of the essential oil extracted from *A. malaccensis* agarwood.

In the most recent study of the antioxidant potential of agarwood, the methanolic extract of an *A. sinensis* leaf was subjected to chromatographic isolation using different elution solvents, starting with water and then increasing the concentration of ethanol [55]. Later, the elutions were tested for their antioxidant potential using ABTS and FRAP radical scavenging assays. As a result, the highest antioxidant potential was determined from the 30% ethanol elution with EC_50_ values for the ABTS and FRAP assays of 366.0 ug/mL and 833.0 ug/mL, respectively. The crude total extracts reported EC_50_ values for the ABTS and FRAP assays of 542.0 ug/mL and 899.0 ug/mL, respectively. Further analysis using tandem mass spectrometry revealed that the 30% ethanol elution fraction is high in antioxidant molecules, particularly mangiferin [55]. These findings further support mangiferin as the main bioactive compound in the antioxidant properties of agarwood leaf extracts.

Strong antioxidant activity was reported, particularly from the ethanol and acetone extractions of *A. malaccensis*, *A. sinensis*, and *G. versteegii* leaves. Mangiferin was found to be the main active compound for antioxidant potential. The antioxidant potential of artificial agarwood is approaching the level of natural agarwood. In conclusion, these studies reviewed and demonstrated significant antioxidant capacities using different parts, compounds, and species of agarwood.

### 4.2. In Vivo Anti-Diabetic Findings of Agarwood

**Figure 3:**
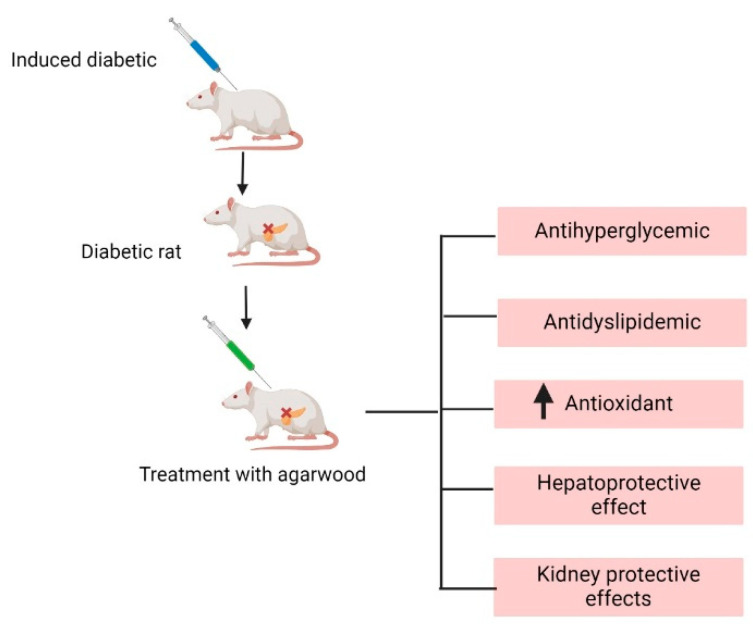
Overview of the in vivo anti-diabetic mechanisms of agarwood.

Several in vivo studies have explored the antidiabetic potential of agarwood and agarwood-producing plants. This research explored the potential of pure extracts as well as synergistic effects with different plant products. Figure 3 summarises the overview of the in vivo anti-diabetic mechanism of agarwood with multiple pathways involved.

First, Pranakhon et al. (2011) demonstrated the hypoglycaemic effect of *A. sinensis* leaf extracts using streptozotocin-induced diabetic rats [48]. Hyperglycaemia was induced by an intraperitoneal injection of 45 mg/kg bodyweight of STZ. After seven days, treatment was given daily for one week by subcutaneous injections of 4 U/kg/day of insulin or the oral administration of 1 g/kg bodyweight/day of 0.15 g/mL of *A. sinensis* leaf extracts. A reduced fasting blood glucose was reported in the methanol and water extract rats of 54% and 40%, respectively. The insulin control group reported a 73% fasting blood glucose reduction. Further analysis also showed that the methanol and water extracts increased glucose uptake by the rat adipocytes by 172% and 176%, similar to the effect of a 1.5 nM insulin treatment. These results showed that agarwood leaves have a hypoglycaemic effect in diabetic rats by increasing glucose uptake from the blood into the adipocytes [48].

In 2015, Pranakhon et al. continued to analyse further the hypoglycaemic effects of *A. sinensis* leaf extracts [49]. Chromatographic isolation revealed iriflophenone 3-C-β-glucoside as the main compound isolated from the *A. sinensis* leaf extracts. Streptozotocin was used to induce diabetes in mice models. The diabetic mice models then received a daily oral administration of 1 g/kg of *A. sinensis* extracts or 0.47 g/kg of iriflophenone 3-C-β-glucoside. The treatment continued for 3 weeks. The iriflophenone 3-C-β-glucoside lowered the glucose level by 46.4%, whereas the crude *A. sinensis* leaf extracts lowered the glucose level by 40.3%. Glucose analysis using rat adipocytes also showed that iriflophenone 3-C-β-glucoside increased glucose uptake by 153%, whereas the crude *A. sinensis* leaf extracts increased glucose uptake by 152%. These findings suggest that iriflophenone 3-C-β-glucoside is the active compound contributing to the hypoglycaemic effect of *A. sinensis* leaves [49].

A study in 2011 further investigated the mechanisms and hypoglycaemic effects of *A. sinensis* leaves using genetically diabetic db/db mice models [53]. The mice received an oral administration of 300 mg/kg of *A. sinensis* extracts in the low-dose group or 600 mg/kg in the high-dose group. After 1 month of treatment, the high-dose group recorded a significant reduction in fasting blood glucose and HbA1c levels. Western blot analysis revealed that the high-dose and rosiglitazone control groups had a higher ratio of phosphorylated AMPK to total AMPK in comparison to the negative control groups. This finding signals the possibility that the hypoglycaemic property effects of *A. sinensis* leaf extracts are due to the activation of AMPK. The study also ran an oral glucose tolerance test (OGTT), which revealed that the extracts protected the mice from sugar spikes by increasing insulin sensitivity. Weight analysis also showed that the extracts did not cause weight gain, unlike the side effects caused by the conventional drug type of thiazolidinediones [53]. These findings further emphasised the potentials and mechanisms of the hypoglycaemic properties of *A. sinensis* leaf extracts.

Two studies were conducted to investigate the synergistic therapeutic effect of *A. lignum* fermented with green tea against diabetes [51,52]. High-fat-fed mice and genetically type 2 diabetes db/db mice were used as diabetic animal models. *A. lignum* was described as the stem of an agarwood plant, *A. agallocha*. The process of producing agarwood-fermented green tea was started by mixing dried green leaves and *A. lignum* powder using a ratio of 49:1. The mixtures were then subjected to several steps of fermentation. In both studies, the mice were administered up to 400 mg/kg of agarwood-fermented green tea orally. The findings reported that in both the high-fat-fed mice and db/db mice, there were significantly stronger hypoglycaemic effects in the agarwood-fermented green tea group than in the conventional green tea group. Histopathological studies reported that agarwood-fermented green tea treatment attenuated the hypertrophy or hyperplasia of pancreatic islet cells by normalising the ratio of insulin-producing pancreatic cells and glucagon-producing cells. Further analysis reported that agarwood-fermented green tea significantly reduced the effect of high-fructose, high-fat diets on the levels of key enzymes involved in glucose metabolism, such as glucokinase, glucose-6-phosphatase, and phosphoenolpyruvate carboxykinase, which may explain the mechanism of hypoglycaemic effects. Other than that, agarwood-fermented green tea also attenuated the increase in lipids and weight gain in both obese and diabetic mice. Agarwood-fermented green tea groups reported a marked decrease in lipid profiles, serum leptin, and faecal lipid content, as well as attenuated adipose accumulation. Further analysis also showed that agarwood-fermented green tea reduced the levels of pancreatic digestive enzymes in the mice and reduced the number of pancreatic zymogen granules. The authors presumed the product’s ability to inhibit the release and activity of pancreatic lipid-digesting enzymes may be the mechanism explaining the anti-dyslipidaemia effects. Histopathological analysis, along with kidney and liver serum profiles, showed that agarwood-fermented green tea had stronger hepatoprotective and nephroprotective effects against diabetic hepatopathy and nephropathy. Both studies also reported stronger anti-radicals and antioxidant potential in agarwood-fermented green tea groups [51,52]. These findings summarise the synergistic effect of agarwood and green tea against diabetes due to its hypoglycaemic, anti-obesity, anti-dyslipidaemia, kidney protective, liver protective, and antioxidant effects.

Another study used streptozotocin-nicotinamide-induced type 2 diabetic mice to illustrate the hypoglycaemic effect of *A. crassna* leaf extracts. The mouse models were induced with 150 mg/kg of streptozotocin and 120 mg/kg of nicotinamide. After 1 month of intragastric intervention with 1000 mg/kg *A. crassna*, blood glucose levels were reduced by 86%. Thus, this study showed the hypoglycaemic potential of *A. crassna* leaf extracts in diabetic mouse models [50].

The most recent in vivo study investigated the synergistic anti-diabetic effect of *Portulaca oleracea* and *A. malaccensis* leaf extracts using high-fructose, high-fat diet rats [54]. The rats were fed a diet containing 60% fructose and fat calories for 70 days to induce insulin resistance. The treatment group received an oral administration of 400 mg/kg/day of *P. oleracea* or 200 mg/kg/day of *A. malaccensis,* or both. The treatment continued for 1 month. The treatment groups showed attenuated weight gain, decreased blood glucose levels, and lipid peroxidation markers. The treatment also reported improvement in lipid, renal, and liver profiles. However, no synergistic effects were reported from the combination of the *P. oleracea* and *A. malaccensis* groups [54].

The findings from these studies elucidated the antidiabetic potentials of various *Aquilaria* extracts, including *A. sinensis*, *A. crassna*, and *A. malaccensis,* as well as the bark of *A. agallocha,* also called *A. lignum*. Considering the results from these studies, the agarwood dosage range of 200–1000 mg/kg appears to be effective for achieving significant hypoglycaemic effects and improving insulin sensitivity in mice models. Future researchers may start with a low dose of 200 mg/kg to assess the initial effects and safety, a medium dose of 400 mg/kg, and a high dosage of 1000 mg/kg to determine if further benefits can be achieved without adverse effects. This dosage range balances efficacy and safety, as seen in the studies, and allows for a comprehensive evaluation of agarwood’s therapeutic potential in diabetes mice models. The antidiabetic effects of agarwood and agarwood-producing plants are demonstrated in the in vivo studies by lowering blood glucose levels, improving insulin sensitivity, and providing antioxidant benefits. Therefore, future research should focus on clinical studies to confirm the efficacy of these products.

## 5. Limitations of the Study

This review paper has certain limitations. Some information could not be fully retrieved from the original articles. The use of various substances, such as ethanol or methanol in extraction, essential oils, and emulsions, makes it challenging to draw precise conclusions. Additionally, different studies utilised various parts of the agarwood tree, such as the fruit bark, stem bark, or leaves, complicating the assessment of its effectiveness against diabetes-related enzymes. Since agarwood is native to specific regions, the species studied may vary by country, making direct comparisons difficult. Furthermore, the limited number of the selected studies restricts the ability to fully determine agarwood’s effectiveness.

## 6. Future Research

The current review presents the potential of agarwood as an anti-diabetic agent. With the advancement in technology, it makes it possible for us to identify the active compound of agarwood. The improvement of agarwood’s isolation and bioactive characterisation is essential for a better understanding of the mechanisms of anti-diabetic action, including insulin sensitivity, glucose uptake, and lipid metabolism. From the bioactive compound, researchers could explore developing functional food products incorporating agarwood extracts, focusing on palatability and bioavailability. The nutritional profile of these functional foods could be analysed to further study the anti-diabetic properties of agarwood-infused foods. We also suggest investigating the effects of anti-diabetic properties when combining agarwood with other herbal remedies that have been known for anti-diabetic properties to evaluate their synergistic effects.

This review has shown the anti-diabetic properties of agarwood using an animal model. Future research could conduct long-term animal studies to assess the impact of prolonged agarwood supplementation on blood glucose levels and insulin sensitivity. The impact of agarwood on diabetes complications such as neuropathy, retinopathy, and nephropathy could be further investigated using a diabetic animal model.

Further research is needed to elucidate its mechanisms, efficacy, and safety in clinical settings. From our scoping, no clinical trials have been conducted using agarwood to study its anti-diabetic effect. In the future, researchers could conduct a small-scale pilot study to assess the safety and efficacy of agarwood in diabetic patients. Later, randomised controlled trials (RCTs) could be designed to evaluate the efficacy of agarwood in lowering blood glucose levels compared to the standard glucose-lowering agents in diabetic patients. It is interesting to study how genetic, dietary, and lifestyle factors affect the efficacy of the anti-diabetic properties in agarwood in different populations. By addressing existing research gaps, exploring holistic approaches, and considering their use in functional foods, future research can contribute significantly to diabetes management strategies complementary to the current diabetes management.

## 7. Conclusions

Agarwood-producing plants, particularly agarwood and leaf extracts, possess promising potential as alternative or supplementary treatments for diabetes. The crude extracts, as well as isolated bioactive compounds such as polyphenols, phenylethyl chromone, and sesquiterpenoids, have demonstrated significant α-glucosidase, lipase, and α-amylase inhibition, and strong antioxidant capacity. Overall, *A*. *malaccensis* shows great promise for inhibiting α-glucosidase and α-amylase and promoting antioxidant activity, as well as stimulating adiponectin production, highlighting its potential therapeutic applications in diabetes management. The main bioactive compounds identified include phenylethylchromone and polyphenols such as genkwanin and mangiferin. Diabetic animal models treated with agarwood plants showed significant hypoglycaemic, anti-obesity, anti-dyslipidaemia, and organ protection effects. However, from our scoping and literature search, no clinical trials on agarwood in relation to diabetes have been performed. Although these findings suggest that agarwood and agarwood-producing plants could act as valuable agents in managing diabetes, further clinical trials are warranted.

## Figures and Tables

**Table 1:** The effect of agarwood on diabetes in in vitro studies.

No.	References	Country	Plant Species	Plant Parts	Extraction Solvent	Key Findings
1	[40]	China	*A. sinensis*	Agarwood chips	95% Ethanol	2-(2-phenyl ethyl) chromone derivatives were isolated from *A. sinensis* and were subjected to an α-glucosidase inhibition assay. Significant inhibitions were detected from three derivatives, namely, 6,7-dimethoxy-2-[2-(4-hydroxyphenyl) ethyl] chromone, 6,7-dihydroxy-2-[2-(4-methoxyphenyl) ethyl] chromone, and 6-hydroxy-2-[2-(3-methoxy-4-hydroxyphenyl) ethyl] chromone with IC_50_ values of 48.953, 15.655, and 16.318 μg/mL, respectively. Acarbose was used as the positive control (IC_50_ = 632.688 μg/mL).
2	[42]	Indonesia	*A. filaria*	Agarwood chips	95% ethanol	2-(2-phenyl ethyl) chromone derivatives were isolated from *A. filaria* and were subjected to an α-glucosidase inhibition assay. The 21 compounds isolated reported α-glucosidase inhibition with IC_50_ values ranging from 2.256 ± 0.085 to 44.977 ± 0.984 μg/mL. Acarbose and genistein were used as controls, 479.939 ± 2.130 μg/mL and 2.243 ± 0.027 μg/mL, respectively. 6,7-dihydroxy-2-(2-phenyl ethyl) chromone was the most potent α-glucosidase inhibitor with an IC_50_ value of 2.256 ± 0.085 μg/mL.
3	[45]	Laos	*A. malaccensis*	Agarwood chips	70% Methanol	2-(2-phenyl ethyl) chromone derivatives were isolated from *A. malaccensis* and investigated for their effects on adiponectin production. 2-(2-phenyl ethyl) chromone derivatives promoted adiponectin production during adipogenesis in a bone marrow mesenchymal stem cell model. The most potent enhancers of adiponectin production were identified as 6-methoxy-2-(2-phenyl ethyl) chromone and 7-methoxy-2-(2-phenyl ethyl) chromone.
4	[30]	Indonesia	*G. versteegii*	Agarwood chips	Methanol and Acetone	Several wood extracts and extraction solvents of *G. versteegii* were tested for their α-glucosidase inhibitory activity and antioxidant potential. Acetone extracts from infected wood of *G. versteegii* had the highest DPPH antioxidant activity with the IC_50_ value of 65.62 μg/mL. Quercetin was used as a control (8.59 ug/mL). Acetone agarwood extracts had the highest α-glucosidase inhibitory activity with an IC_50_ value of 53.46 μg/mL. Quercetin was used as a control (5.34 ug/mL).
5	[46]	China	*A. sinensis*	Leaves	70% Ethanol	Compounds were isolated from the ethanolic extracts of *A. sinensis* leaves and screened for α-glucosidase inhibitory activity. Compounds were isolated using activity-directed fractionation and a purification process. Mangiferin and iriflophenone 2-O-a-L-rhamnopyranoside, the significant components in the leaves of *A. sinensis*, showed potent inhibition with IC_50_ values of 126.5 ± 17.8 and 143.7 ± 10.6 ug/mL, respectively. Acarbose was used as a control (372.0 ± 37.8 ug/mL).
6	[39]	China	*A. sinensis* and *A. crassna*	Agarwood chips	95% Ethanol	Artificial and wild agarwood extracts were investigated for their antioxidant potential and α-glucosidase inhibition. Wild agarwood demonstrated more substantial antioxidant potential, followed by artificial *A. sinensis* agarwood. IC_50_ values of the α-glucosidase inhibition for wild agarwood, artificial *A. sinensis*, and artificial *A. crassna* were reported at 156.400 ug/mL, 292.000 ug/mL, and 343.000 ug/mL, respectively.
7	[47]	Malaysia	*A. malaccensis*, *A. subintegra*, and *A. sinensis*	Leaves	100% Ethanol, 70% Ethanol, Water, and Hexane	Several leaves of the *Aquilaria* spp. were extracted using various solvents and drying methods. The extracts were screened and compared for their α-glucosidase inhibition and DPPH radical scavenging activity. Oven-dried *A. malaccensis* leaves extracted using 70% ethanol showed the highest inhibition of α-glucosidase and DPPH radicals with IC_50_ values of 0.13 ug/mL and 33.60 ug/mL, respectively. Acarbose was used as a control for the α-glucosidase inhibition test, whereas quercetin was used as the control for the DPPH radical scavenging test. The IC_50_ of the controls was 0.65 ug/mL and 20.75 ug/mL, respectively.
8	[33]	Malaysia	*A. subintegra* and *A. malaccensis*	Leaves and Barks	Hexane, Dichloromethane, and Methanol	Using several extraction solvents, 12 extracts from the leaves and barks of *A. subintegra* and *A. malaccensis* were screened for anti-lipase activity. At 100 ug/mL, *A. malaccensis* bark extracted using dichloromethane showed the most potent lipase inhibition with 91% inhibition.
9	[44]	Laos	*A. crassna*	Agarwood chips	95% Ethanol	Four new derivatives of bi-2-(2-phenyl ethyl) chromone were isolated from the *A. crassna* extracts, which were named crassin A, crassin B, crassin C, and crassin D. α-glucosidase inhibition screening was conducted. None of the derivatives showed significant α-glucosidase inhibition activity.
10	[41]	China	*A. sinensis*	Agarwood chips	95% Ethanol	Four new derivatives of bi-phenyl ethyl chromones were isolated from the *A. sinensis* extracts, which were (5S,6R,7S,8R)-2-[2-(4-Methoxyphenyl)ethyl]-5,6,7-trihydroxy-5,6,7,8-tetrahydro-8-{6-methoxy-2-[2-(3‴-methoxy-4‴-hydroxyphenyl)ethyl]chromonyl-7-oxy}chromone, (5S,6R,7S,8R)-2-[2-(4-Methoxyphenyl)ethyl]-5,6,7-trihydroxy-5,6,7,8-tetrahydro-8-{2-[2-(4‴-methoxyphenyl)ethyl]chromonyl-6-oxy}chromone, 5S,6R,7S,8R)-2-(2-Phenylethyl)-5,6,7-trihydroxy-5,6,7,8-tetrahydro-8-[2-(2-phenyl ethyl)chromonyl-6-oxy]chromone, and (5R,6R,7R,8S)-2-(2-Phenylethyl)-5,6,7-trihydroxy-5,6,7,8-tetrahydro-8-[2-(2-phenyl ethyl)chromonyl-6-oxy]chromone. α-glucosidase inhibition screening was conducted. None of the derivatives showed significant α-glucosidase inhibition activity.
11	[43]	Indonesia	*A. filaria*	Agarwood chips	95% Ethanol	Six 8,12-epoxyguaiane sesquiterpenes were isolated from *A. filaria* agarwood extracts. The extracts were then screened for α-glucosidase inhibitory activity. The most potent α-glucosidase inhibitor was reported from guaianolide with an IC_50_ value of 53.332 ± 2.273 μg/mL. Acarbose was used as a control (IC_50_ = 479.939 ± 2.130 μg/mL).
12	[38]	Thailand	*A. crassna*	Leaves	95% Ethanol	Mangiferin was determined as the active metabolite of *A. crassna* leaf extracts. Mangiferin and crude extracts of *A. crassna* leaves were screened for α-glucosidase inhibition and antioxidant activity. α-glucosidase inhibition of *A. crassna* extracts and mangiferin were reported with IC_50_ values of 184.000 ± 3.200 μg/mL and 571.400 ± 4.400 μg/mL, respectively. Acarbose was used as a control (IC_50_ = 17,394.700 ± 18.9 μg/mL). A DPPH radical scavenging assay, nitric oxide scavenging assay, and superoxide scavenging assay showed that *A. crassna* extracts and mangiferin were significantly antioxidant. Mangiferin reported higher antioxidant potential than the crude extracts, suggesting its role as the active compound.
13	[27]	Malaysia	*A. malaccensis* and *A. subintegra*	Leaves	Water	Various particle sizes of *A. subintegra* and *A. malaccensis* leaves were extracted and screened for lipase inhibition. Highest inhibition was reported using the extracts obtained from the smallest particle size, 250 μm, with *A. subintegra* leaves being the most potent at 82% lipase inhibition. Kinetic study revealed the mode of lipase inhibition from *A. subintegra* and *A. malaccensis* leaves was determined as mixed inhibition that mimics noncompetitive binding.
14	[35]	Thailand	*Aquilaria* spp.	Agarwood chips	Ethyl ether	Sesquiterpenoid derivatives were isolated from unspecified *Aquilaria* spp. and were subjected to an α-glucosidase inhibition assay. Aquilarene D and Aquilarene E reported significant inhibition against α-glucosidase with IC_50_ values of 57.460 and 522.104 μg/mL. Acarbose was used as a control (IC_50_ = 781.176 μg/mL).
15	[31]	Indonesia	*G. versteegii*	Fruit bark, Stem bark, and Leaves	Methanol	Extracts obtained from various parts of *G. versteegii* were tested for their α-glucosidase inhibitory activity and antioxidant potential. Leaf extracts of *G. versteegii* had the highest potency against α-glucosidase activity with an IC_50_ value of 55.01 ± 2.4 ug/mL. Quercetin was used as a control (IC_50_ = 5.34 ± 0.2 ug/mL). Leaf extracts of *G. versteegii* showed the highest antioxidant potential using a DPPH radical scavenging assay with an IC_50_ value of 32.89 ± 2.7 ug/mL. Quercetin was used as a control (IC_50_ = 8.59 ± 0.3 ug/mL).
16	[34]	Thailand	*Aquilaria* spp.	Agarwood chips	Ethyl ether	Sesquiterpenoids derivatives were isolated from unspecified *Aquilaria* spp. and were subjected to an α-glucosidase inhibition assay. Significant α-glucosidase inhibition was reported from agarozizanol E, jinkohol I, jinkohol II, and isokhusenol, with IC_50_ values ranging from 27.308 μg/mL to 109.274 μg/mL. Acarbose was used as a control (IC_50_ = 479.939 μg/mL). A kinetic study reported an uncompetitive mode of inhibition against α-glucosidase activity.
17	[29]	China	*A. sinensis*	Leaves	Not stated	Several benzophenone O-glycosides were synthesised from *A. sinensis* leaf extracts and screened for their potential to inhibit α-glucosidase activity. Significant α-glucosidase inhibition was reported using 4,4′,6-O-Trihydroxylbenzophenone 2-O-a-D-mannopyranosyl-(1-4)-a-L-rhamnoside and 2,4′,6-O-Trihydroxylbenzophenone 4-O-a-D-mannopyranosyl-(1-4)-a-L-rhamnoside, they reported IC_50_ values of 93.713 ± 7.721 μg/mL and 116.710 ± 13.276 μg/mL, respectively. Acarbose was used as a control (IC_50_ = 367.540 ± 32.086 μg/mL).
18	[32]	Vietnam	*A. crassna*	Branches	Methanol	Several polyphenols, namely, iriflophenone 3,5-C-β-D-diglucoside, iriflophenone 3-C-β-D-glucoside, mangiferin, iriflophenone 2-O-α-rhamnoside, genkwanin 5-O-β-primeveroside, genkwanin 4′-methyl ether 5-O-β-primeveroside, and genkwanin, were isolated from *A. crassna* branches and screened for their potential to inhibit α-glucosidase activity. Genkwanin was found to have the highest inhibition of α-glucosidase activity with an IC_50_ value of 6.822 ug/mL. Acarbose was used as a control (IC_50_ = 50.05 ug/mL).
19	[28]	India	*A. malaccensis*	Agarwood chips	Water	Essential oil extracted from *A. malaccensis* agarwood was screened for its α-amylase inhibitory and antioxidant activity. The IC_50_ value for the α-amylase inhibitory assay was reported at 30.78 ± 0.0018 μL/mL. Acarbose was used as a control (IC_50_ = 44.77 ± 0.0006 μL/mL). DPPH, 2,2′-Azino-bis (3-ethylbenzothiazoline-6-sulfonic acid) (ABTS) radical scavenging and metal chelating assays were conducted to determine its antioxidant activity. The IC_50_ values reported were 40.14 ± 0.0192, 76.95 ± 0.0090, and 26.96 ± 0.0244 μL/mL, respectively. α-tocopherol was used as a control for the DPPH and ABTS assays, with IC_50_ values of 18.53 ± 0.0141 and 61.93 ± 0.0018 μL/mL, respectively. Ethylenediaminetetraacetic acid (EDTA) was used as a control for the metal chelating assay with an IC_50_ value of 19.52 ± 0.0192 μL/mL.
20	[37]	China	*A. sinensis*	Agarwood chips	Ethanol	Artificial and wild agarwood extracts were investigated for their antioxidant potential and α-glucosidase inhibition. Showing a similar trend from 2021, IC_50_ values of the α-glucosidase inhibition for wild agarwood were higher compared to artificial *A. sinensis* with IC_50_ values of 0.147 ug/mL and 0.212 ug/mL, respectively. Wild agarwood reported higher antioxidant capacity than artificial agarwood via DPPH and ABTS assays. However, the differences were not statistically significant (*p* > 0.05).
21	[36]	Indonesia	*A. malaccensis*	Leaves	Ethanol	The synergistic effect of *A. malaccensis* leaves and *Cinnamomum burmannii* bark extracts on α-glucosidase and α-amylase was investigated. 750:250 ug/mL *C. burmannii* and *A. malaccensis* extracts reported increased inhibition of α-glucosidase and α-amylase compared to using the extracts individually.
22	[55]	China	*A. sinensis*	Leaves	Methanol	*A. sinensis* leaf extracts were isolated using different elution solvents, starting with water, 30% ethanol, 50% ethanol, 75% ethanol, and 95% ethanol. The eluates were tested for α-glucosidase inhibitory and antioxidant potential. 75% ethanol eluates reported the highest α-glucosidase inhibition with EC_50_ value 844 ug/mL. Acarbose was used as a control (EC_50_ = 183 ug/mL). 30% ethanol eluates reported the highest antioxidant capacity through ABTS and ferric reducing antioxidant power (FRAP) radical scavenging assays. IC_50_ values were 366 ug/mL and 833 ug/mL, respectively.

**Table 2:** The effect of agarwood on diabetes in in vivo studies.

No.	References	Country	Plant Species	Part of Plant	Extraction Solvent	Experimental Model	Main Outcomes
1	[48]	Thailand	*A. sinensis*	Leaves	Water and Methanol	Model: male Sprague–Dawley rats. Induction of disease: streptozotocin. Treatment: 1 g/kg of extracts. Control: 4 U/kg/day of insulin. Treatment duration: 7 days.	*A. sinensis* extracts reduced fasting blood glucose up to 54%, comparable to insulin control at 73%. *A. sinensis* extracts enhanced glucose uptake by adipocytes by up to 176%, comparable to insulin control at 166%.
2	[53]	China	*A. sinensis*	Leaves	95% Ethanol	Model: db/db mice. Induction of disease: genetically diabetic mice. Treatment: 300–600 mg/kg of extracts. Control: 5 mg/kg of rosiglitazone. Treatment duration: 1 month.	*A. sinensis* extracts reduced hyperglycaemia in oral glucose tolerance tests and glycosylated haemoglobin (HbA1C) levels, implying that the treatment managed to reduce insulin resistance. *A. sinensis* extracts also attenuated weight gain, which is a side effect of thiazolidinediones. Western blot analysis revealed that the mechanism of the hypoglycaemic effect by *A. sinensis* may be related to the activation of AMP-activated protein kinase (AMPK).
3	[49]	Thailand	*A. sinensis*	Leaves	Methanol	Model: imprinting control region mice. Induction of disease: streptozotocin. Treatment: 0.1–1 g of extracts or 0.047–0.47 g of iriflophenone 3-C-β-glucoside. Control: 8 U/kg/day of insulin. Treatment duration: 3 weeks.	Iriflophenone 3-C-β-glucoside was identified as the main constituent in *A. sinensis* extracts at 3.17%. *A. sinensis* extracts and iriflophenone 3-C-β-glucoside reduced blood glucose levels by 40.3% and 46.4%, respectively. *A. sinensis* extracts and iriflophenone 3-C-β-glucoside enhanced glucose uptake by adipocytes up to 152% and 153%, respectively. Iriflophenone 3-C-β-glucoside was suggested as the main active compound for the hypoglycaemic effect in *A. sinensis* extracts.
4	[51]	Korea	*A. lignum (A. agallocha)*	Agarwood	Water	Model: female Specific Pathogen-Free/Virus Antibody Free Crj: CD-1 (SPF/VAF CrljOri:CD1) Imprinting Control Region (ICR) mice. Induction of disease: high-fat diet. Treatment: 100–400 mg/kg of agarwood-fermented green tea or 400 mg/kg of green tea. Control: 250 mg/kg of metformin or 10 mg/kg of simvastatin. Treatment duration: 84 days.	Agarwood-fermented green tea improves the anti-diabetic potential of normal green tea by enhancing its antihyperglycemic, antidyslipidemia, antioxidant, hepatoprotective, and kidney protective effects. Agarwood-fermented green tea attenuated the decrease in glucokinase and the increase in glucose-6-phosphatase and phosphoenolpyruvate carboxykinase, which are liver enzymes related to glucose homeostasis.
5	[52]	Korea	*A. lignum (A. agallocha)*	Agarwood	Water	Model: female C57BL/6NCrljOri mice and db/db mice. Induction of disease: genetically diabetic mice. Treatment: 100–400 mg/kg of agarwood-fermented green tea or 400 mg/kg of green tea. Control: 250 mg/kg of metformin. Treatment duration: 84 days.	Synergistic effects of agarwood and green tea were reported by enhanced anti-obesity, hypoglycaemic, hypolipidemic, anti-hepatopathy, anti-nephropathy, and antioxidant effects.
6	[50]	Thailand	*A. crassna*	Leaves	Water	Model: Male ddY mice. Induction of disease: streptozotocin and nicotinamide. Treatment: 5–1000 mg/kg of extracts. Control: 5 mg/kg of glibenclamide. Treatment duration: 28 days.	*A. crassna* extracts treatment managed to attenuate blood glucose spiking significantly and promoted a hypoglycaemic effect of up to 86% compared to the control (*p* < 0.01). *A. crassna* extracts also presented anti-α-glucosidase effects with an IC_50_ value of 36.3 ug/mL and an antioxidative effect with an IC_50_ value of DPPH inhibition determined at 34.6 ug/mL. Enzyme-kinetic analysis revealed the uncompetitive inhibition of α-glucosidase by *A. crassna* extracts.
7	[54]	Algeria	*A. malaccensis*	Stem barks	70% Methanol	Model: Female albino rats. Induction of disease: high-fructose, high-fat diet. Treatment: 200 mg/kg of agarwood extracts or 400 mg/kg of *P. oleracea* extracts or both. Control: 300 mg/kg of metformin. Treatment duration: 1 month.	*A. malaccensis* extracts significantly attenuated the effect of insulin resistance induced by a high-fructose, high-fat diet in mice Hypoglycaemic and anti-dyslipidaemia were reported by reduced blood glucose and serum lipid profile. *A. malaccensis* reduced liver and kidney damage caused by insulin resistance by improving kidney and liver function markers. *A. malaccensis* showed antioxidant potential by significantly reducing the lipid peroxidation marker malondialdehyde. However, no synergistic effect was found between *A. malaccensis* and *P. oleracea.*

## Supplementary Materials

The following supporting information can be downloaded at: https://www.mdpi.com/article/10.3390/ph17111548/s1.

## Abbreviations

ABTS	2,2′-Azino-bis (3-ethylbenzothiazoline-6-sulfonic acid)
AMPK	AMP-activated protein kinase
CITES	Convention of International Trade in Endangered Species of Wild Fauna and Flora
db/db mice	Diabetic and obese mice
DPP-4	Dipeptidyl Peptidase-4
DPPH	2,2-Diphenyl-1-picrylhydrazyl
EC50	Half maximal effective concentration
EDTA	Ethylenediaminetetraacetic acid
FBG	Fasting Blood Glucose
FRAP	Ferric Reducing Antioxidant Power
GLP-1	Glucagon-Like Peptide-1
HbA1c	Glycosylated Haemoglobin
IC50	Half maximal inhibitory concentration
ICR mice	Imprinting Control Region mice
IDF	International Diabetes Federation
IR	Insulin Resistance
MeSH	Medical Subject Heading
PICO	Population, Intervention, Comparison, Outcome
PPAR	Peroxisome Proliferator-Activated Receptor
PRISMA	Preferred Reporting Items for Systematic reviews and Meta-Analyses
RCT	Randomised clinical trials
SGLT2	Sodium-Glucose Cotransporter-2
SPF/VAF CrljOri ICR mice	Specific Pathogen-Free/Virus Antibody Free Crj: CD-1 ICR mice
