# Supplementary material for: The Potential Therapeutic Use of Agarwood for Diabetes: A Scoping Review
Source: Pharmaceuticals (Basel). 2024 Nov 18;17(11):1548. doi: 10.3390/ph17111548 (PMC11597494; doi:10.3390/ph17111548)
Supplement: Supplementary file 1 [file pharmaceuticals-17-01548-s001.zip › pharmaceuticals-3230862-supplementary/Supp Material S1 - Detailed search terms for database browsing..docx]

S1: Detailed search terms for database browsing.

| Databases | Search Terms | Hits |
| --- | --- | --- |
| Pubmed | (((((((((((diabetes[MeSH Terms]) OR (Diabetes Mellitus, Type 2[Title/Abstract])) OR (type 2 diabetes mellitus[Title/Abstract])) OR (diabetes type 2[Title/Abstract])) OR (T2DM[Title/Abstract])) OR (diabetes Type II[Title/Abstract])) OR (diabetes[Title/Abstract])) OR (glucosidase[Title/Abstract])) OR (lipase[Title/Abstract])) OR (insulin resistance[Title/Abstract])) OR (amylase[Title/Abstract])) AND ((((((((((((aquilaria[MeSH Terms]) OR (Agarwood[Title/Abstract])) OR (aloeswood[Title/Abstract])) OR (eaglewood[Title/Abstract])) OR (gharuwood[Title/Abstract])) OR (oud[Title/Abstract])) OR (oudh[Title/Abstract])) OR (*Aquilaria* malaccensis[Title/Abstract])) OR (*Aquilaria* sinensis[Title/Abstract])) OR (*Aquilaria* agallocha[Title/Abstract])) OR (*Aquilaria* crassna[Title/Abstract])) OR (*Gyrinops*[Title/Abstract])) | 71 |
| Cochrane Library | #1 MeSH descriptor: [Diabetes Mellitus] explode all trees  #2 (diabetes):ti,ab,kw  #3 (Diabetes Mellitus, Type 2):ti,ab,kw  #4 (type 2 diabetes mellitus):ti,ab,kw  #5 (diabetes type 2):ti,ab,kw  #6 (diabetes Type II):ti,ab,kw  #7 (T2DM):ti,ab,kw  #8 (insulin resistance):ti,ab,kw  #9 (glucosidase):ti,ab,kw  #10 (lipase):ti,ab,kw  #11 (amylase):ti,ab,kw  #12 #1 OR #2 OR #3 #4 OR #5 OR #6 OR #7 OR #8 OR #9 OR #10 OR #11  #13 MeSH descriptor: [Thymelaeaceae] explode all trees  #14 (agarwood):ti,ab,kw  #15 (oud):ti,ab,kw  #16 (oudh):ti,ab,kw  #17 (gharuwood):ti,ab,kw  #18 (aloeswood):ti,ab,kw  #19 (eaglewood):ti,ab,kw  #20 ("*Aquilaria* crassna"):ti,ab,kw  #21 ("*Aquilaria* malaccensis"):ti,ab,kw  #22 ("*Aquilaria* sinensis"):ti,ab,kw  #23 ("*Aquilaria* agallocha"):ti,ab,kw  #24 (*Gyrinops*):ti,ab,kw  #25 #13 OR #13 OR #14 OR #15 OR #16 OR #17 OR #18 OR #19 OR #20 OR #21 OR #22 OR #23 OR #24  #26 #12 AND #25 | 4 |
| Scopus | ( ( ( TITLE-ABS-KEY ( diabetes ) ) OR ( TITLE-ABS-KEY ( t2dm ) ) OR ( TITLE-ABS-KEY ( "diabetes type ii" ) ) OR ( TITLE-ABS-KEY ( "diabetes type 2" ) ) OR ( TITLE-ABS-KEY ( "diabetes mellitus type 2" ) ) OR ( TITLE-ABS-KEY ( "type 2 diabetes mellitus" ) ) ) OR ( TITLE-ABS-KEY ( "insulin resistance" ) ) OR ( TITLE-ABS-KEY ( glucosidase ) ) OR ( TITLE-ABS-KEY ( lipase ) ) ) AND ( ( ( TITLE-ABS-KEY ( agarwood ) ) OR ( TITLE-ABS-KEY ( oud ) ) OR ( TITLE-ABS-KEY ( oudh ) ) OR ( TITLE-ABS-KEY ( gharuwood ) ) OR ( TITLE-ABS-KEY ( aloeswood ) ) OR ( TITLE-ABS-KEY ( eaglewood ) ) OR ( TITLE-ABS-KEY ( "*Aquilaria* crassna" ) ) OR ( TITLE-ABS-KEY ( "*Aquilaria* malaccensis" ) ) OR ( TITLE-ABS-KEY ( "*Aquilaria* sinensis" ) ) OR ( TITLE-ABS-KEY ( "*Aquilaria* agallocha" ) ) OR ( TITLE-ABS-KEY ( *Aquilaria* ) ) ) OR ( TITLE-ABS-KEY ( *Gyrinops* ) ) ) | 100 |
| Total | | 175 |
